# Fluorescence Polarization Immunoassay with Modulated Selectivity for Effective Detection of the Agrochemical 4-Chlorophenoxyacetic Acid

**DOI:** 10.3390/bios16060343

**Published:** 2026-06-18

**Authors:** Marya K. Kolokolova, Liliya I. Mukhametova, Boris S. Tupertsev, Anatoly V. Zherdev, Xinxin Xu, Chuanlai Xu, Sergei A. Eremin

**Affiliations:** 1 Faculty of Chemistry, M. V. Lomonosov Moscow State University, Leninsky Gory 1/3, 119991 Moscow, Russia; kolokolovamasha@yandex.ru (M.K.K.); liliya106@mail.ru (L.I.M.); 2 A. N. Bach Institute of Biochemistry, Research Center of Biotechnology of the Russian Academy of Sciences, Leninsky Prospect 33, 119071 Moscow, Russia; zherdev@inbi.ras.ru; 3 N. N. Semenov Federal Research Center for Chemical Physics, Russian Academy of Sciences, Kosygina 4, 119991 Moscow, Russia; btoupersev@gmail.com; 4 State Key Laboratory of Food Science and Technology, School of Food Science and Technology, Jiangnan University, Wuxi 214122, China; xxx89826@163.com (X.X.); xcl@jiangnan.edu.cn (C.X.)

**Keywords:** agrochemicals, chlorophenoxyacetic acids, immunosensering, fluorophores, fluorescence polarization, toxic contaminants, on-site detection, water safety control

## Abstract

4-Chlorophenoxyacetic acid (4-CPA), a synthetic auxin analog, is employed in agriculture both as a plant growth regulator and as a constituent of herbicide formulations. Consequently, the establishment of simple and rapid detection methods is essential for effective environmental monitoring. This study reports the first development of a homogeneous fluorescence polarization immunoassay (FPIA) for the determination of 4-CPA. The monoclonal antibody (M1), raised against 4-CPA, was evaluated as a recognition element. Furthermore, two fluorescently labeled 4-CPA tracers—with ethylenediamine fluorescein thiocarbamate and aminohexylaminocarbonylfluorescein—were synthesized and purified, and their structures were unequivocally confirmed by high-performance liquid chromatography coupled with high-resolution mass spectrometric detection (HPLC-HRMS). Optimal concentrations of monoclonal antibodies and tracers were established, yielding a limit of detection of 1.2 ng/mL. The assay demonstrated a broad dynamic range of 2.3–300 ng/mL and a rapid analysis time of 15 min. Validation via the standard addition method in authentic open water samples resulted in recovery rates of 98–112%. To address the cross-reactivity with the prevalent herbicide 2,4-dichlorophenoxyacetic acid (2,4-D), two novel strategies were devised and successfully implemented. The first approach involves the concurrent execution of two separate FPIAs—one for 2,4-D and one for 4-CPA—followed by the mathematical resolution of two analyte concentrations from the two measured binding values. The second strategy entails the preliminary selective removal of 2,4-D from sample matrices using affinity chromatography columns with immobilized anti-2,4-D antibodies prior to FPIA for 4-CPA. These proposed methodologies appear highly promising for overcoming the inherent limitations of traditional immunoassays when faced with significant cross-reactivity among structurally analogous compounds.

## 1. Introduction

4-Chlorophenoxyacetic acid (4-CPA) is a component of plant growth regulators used to increase fruit set, enhance growth, accelerate ripening, increase yields, and improve the quality of tomatoes, sweet peppers, and eggplants [1,2,3,4]. It promotes faster growth of mulberry and the accumulation of four main flavonoids in its leaves: rutin, chlorogenic acid, isoquercitrin, and astragalin [5] and is used as a growth regulator in the germination of mung beans [6]. 4-CPA is a synthetic auxin analog and has high activity in the activation of the proton pump [7,8]. Like its famous structural analog, the widely applied herbicide 2,4-D (2,4-dichlorophenoxyacetic acid), 4-CPA is a growth stimulant at low doses and a powerful systemic herbicide at high doses. At low concentrations (0.52–2.0 mg/L final concentration in the nutrient medium), 4-CPA promotes callus formation; at higher concentrations, typically when the upper limit is exceeded three to five times (i.e., more than 6–10 mg/L), it acts as an herbicide, leading to irreversible disruption of the plant’s hormonal system and plant death. Furthermore, excessive use of 4-CPA can lead to excessively rapid or, conversely, slow crop growth and even plant death, which undoubtedly impacts the quality of agricultural products [9,10]. 4-CPA exhibits cumulative toxicity in humans, and excessive consumption can cause harm to health, potentially leading to damage to vital organs such as the kidneys and liver [11,12]. Therefore, the development of sensitive methods for detecting 4-CPA is promising for monitoring its residues in food products and water. According to the US Environmental Protection Agency, for example, the maximum permissible concentration of 4-CPA for mung bean sprouts is 200 ppb and for tomatoes—50 ppb [13]. Therefore, rapid and sensitive methods for monitoring this substance are needed.

Instrumental methods of analysis for the 4-CPA determination have proven themselves to be effective [12,14,15,16,17,18]. It has been shown that gas chromatography achieves a limit of detection (LOD) of 10 pg/mL for 4-CPA [16] or 240 pg/mL for gas chromatography with mass spectrometry detection [12]. However, these methods require expensive equipment and highly qualified personnel and can only be performed in a limited row of specially equipped laboratories.

Currently, the immunoassays for agrochemicals remain a promising area [11,19,20,21,22,23,24]. ELISA is a widely used and practical method for determining contaminants, accessible for on-site analysis, characterized by low cost and high specificity [25]. Recent developments include a visual colorimetric immunosensor with a limit of 4-CPA detection of 0.2 ng/mL [26] and an immunochromatographic test for the rapid detection of 4-CPA in bean sprout extracts with a visual limit of detection of 12.5 ng/mL [19]. Another immunoassay used for toxicants detection is the fluorescence polarization immunoassay (FPIA), which is a rapid, sensitive, and homogeneous technique. This method is based on the phenomenon of fluorescence polarization (FP) and measures the degree of fluorescence depolarization that occurs during the rotational diffusion of molecules between the absorption of plane-polarized light and emission. To develop a highly sensitive FPIA, it is necessary to select a pair of immunoreagents: high-affinity antibodies to the antigen and a conjugate of the antigen with a fluorescent label (tracer). FPIA is relatively simple to set up; its protocol consists only in adding aliquots of the tracer and antibody solution to the sample, incubating for several minutes, and measuring the fluorescence polarization signal. FPIA is a homogeneous “mix and measure” method widely used for the detection of low-molecular-weight compounds. During analysis, the degree of polarization of the emitted light reflects the result of competitive binding with antibodies, thereby allowing for a highly accurate determination of the analyte concentration in the sample [27,28,29,30,31]. The aim of this study is to develop a rapid and sensitive FPIA method for the determination of 4-CPA.

Highly sensitive immunoassays require high-affinity immunoreagents. Monoclonal antibodies with high specificity to the target antigen have proven effective in detecting pesticides using immunoassays [32] To develop a rapid FPIA, it is necessary to obtain tracers—conjugates of the antigen with a fluorescent dye—that should efficiently bind to antibodies. For this study, new tracers of 4-CPA with ethylenediamine fluorescein thiocarbamate (EDF, the linker length is two CH_2_ groups) and aminohexylaminecarbonylfluorescein (6-AHF, the linker length is six CH_2_ groups) will be prepared and characterized, since the linker length in the conjugate can affect the sensitivity of the assay [33,34]. In this study, highly specific monoclonal antibodies will be obtained for the detection of low concentrations of 4-CPA. Tracer/antibody immunoreagent pairs will be selected, and a sensitive and selective fluorescence polarization immunoassay will be developed for the rapid, on-site detection of 4-CPA. Antibody cross-reactivity also requires careful consideration, as it may interfere with target antigen detection in the presence of structurally similar analogs. This limitation can be minimized using various approaches. In this study, alternative approaches for the separate detection of 4-CPA in the presence of 2,4-D will be tested and implemented. The first proposed methodology is based on the simultaneous or parallel operation of two different fluorescence polarization immunoassays (FPIAs), each designed to analyze different substances. Specifically, one FPIA is designed to detect and quantify 2,4-dichlorophenoxyacetic acid (2,4-D), while the second FPIA specifically targets 4-chlorophenoxyacetic acid (4-CPA). Both assays are run simultaneously on the same sample. After obtaining two binding response values—one from each FPIA—mathematical processing is applied, such as solving a system of equations based on known cross-reactivity models or calibration models, to determine the individual concentrations of the two analytes present in the sample. This approach allows for differentiation of structurally similar compounds without physical separation prior to detection. The second strategy involves a selective sample pretreatment step prior to FPIA measurement for 4-CPA. In this approach, 2,4-D is first removed from the sample matrix using affinity chromatography columns. These columns are packed with a solid support immobilized with antibodies to 2,4-D, allowing them to specifically capture and retain 2,4-D molecules while allowing other analytes, including 4-CPA, to pass through. Following this selective removal of 2,4-D, the remaining sample—now free of 2,4-D interference—is subjected to FPIA to determine only 4-CPA. This physical separation strategy effectively eliminates the risk of cross-reactivity with 2,4-D in subsequent immunoassays.

Both proposed methods show significant promise as a solution to one of the main problems of traditional immunoassays: their inability to reliably distinguish structurally similar compounds in the presence of significant cross-reactivity. Using either mathematical resolution or affinity-based pre-separation, these approaches improve the specificity and accuracy of analysis in complex sample matrices containing multiple similar analytes.

## 2. Materials and Methods

### 2.1. Reagents

The following reagents were used in this study: N-hydroxysuccinimide (NHS), N,N′-dicyclohexylcarbodiimide (N,N′-DCC), 4-chlorophenoxyacetic acid (4-CPA), 2,4-dichlorophenoxyacetic acid (2,4-D), 3,4-dichlorophenoxyacetic acid (3,4-D), 2,4,5-trichlorophenoxyacetic acid (2,4,5-T), 2-methyl-4-chlorophenoxyacetic acid (MCPA) glyphosate, atrazine and chlorpyrifos (Sigma-Aldrich, St. Louis, MA, USA), monoclonal antibody against glyphosate (M2), monoclonal antibody against 2,4-D (MAb 2,4-D) [29], sodium azide (Serva, Heidelberg, Germany), fluorescein thiosemicarbazide (Fluka, Buchs, Switzerland), sodium sulfate and sodium hydrogen sulfate (Sigma-Aldrich, USA), ethyl acetate (Scharlau, Sentmenat, Spain), chloroform (Khimmed, Moscow, Russia), methanol, acetonitrile, dichloromethane, dimethylformamide (DMF), triethylamine, and HPLC-grade formic acid (Merck, Darmstadt, Germany), BrCN-activated Sepharose 4B (GE Healthcare Bio-Sciences AB, Uppsala, Sweden) Kieselgel 60 F254 plates (Merck, Darmstadt, Germany) were used for thin-layer chromatography (TLC).

### 2.2. Production of 4-CPA Monoclonal Antibodies

The immunogen (BSA tracer) was prepared using the EDC/NHS method [35]. Briefly, 2,4-D (1 mg) and NHS (0.52 mg) were dissolved in 300 μL DMF. Separately, 0.86 mg of EDC was dissolved in 20 μL of 0.1 M MES buffer (pH 9.0). The EDC solution was slowly added to the above mixture under stirring and allowed to react for 6–8 h to activate the carboxyl group. The activated solution was then added dropwise to 0.01 M PBS containing 3 mg BSA and stirred overnight at room temperature. The coating antigen (OVA tracer) was synthesized using the same EDC/NHS procedure, with a hapten-to-protein feeding molar ratio of 60:1. Specifically, 7.6 mg of EDC, 4.6 mg of NHS, 3.3 mg of 2,4-D, and 10 mg of OVA were used. Either the immunogen or the coating antigen products were dialyzed in 0.01 M PBS, pH 7.4, to remove unreacted small molecules. The immunogen or coating antigen was aliquoted for storage at −20 °C before use.

The antibody preparation referred to previous studies [35,36]. Briefly, the immunogen was emulsified with complete Freund’s adjuvant and administered to BALB/c mice in the first immunization (100 μg). In the following four booster immunizations (50 μg), the immunogen was emulsified with incomplete Freund’s adjuvant. The mouse exhibiting the highest serum antibody titer and inhibition rate, as determined by indirect competitive ELISA (ic-ELISA), was selected for subsequent cell fusion. Monoclonal antibodies were produced using classical hybridoma technology, including PEG-mediated fusion of splenocytes with SP2/0 myeloma cells, followed by in vivo antibody production using the ascites method in mice. MAb M1 was purified from the ascites using protein G affinity and aliquoted for storage at −20 °C before use.

All animal experiments strictly complied with the “Regulation for the Administration of Affairs Concerning Experimental Animals” in China and were approved by the Animal Ethics Committee of Jiangnan University.

### 2.3. Testing for Antibodies Specific to 2,4-D

Antibodies to 2,4-D were adsorbed on wells of polystyrene microplates at a concentration of 1 μg/mL in a carbonate-bicarbonate buffer solution (pH 9.0) (100 μL per well). The plates were incubated overnight at +4 °C, washed with 300 μL of PBS containing 0.1% Tween, and blocked with 1% BSA for 3 h at room temperature. The blocking buffer was removed, and the plate was dried overnight at room temperature.

To conduct an experiment to study the cross-specificity of antibodies specific to 2,4-D and 4-CPA, working solutions of 5 ng/mL and 10 ng/mL, respectively, were prepared. Solutions of 4-CPA and 2,4-D were also prepared in bicarbonate buffer pH 8.5 at concentrations of 100, 10, 1, 0.1, 0.01, 0.001, and 0.0001 µg/mL.

**Figure 1:**
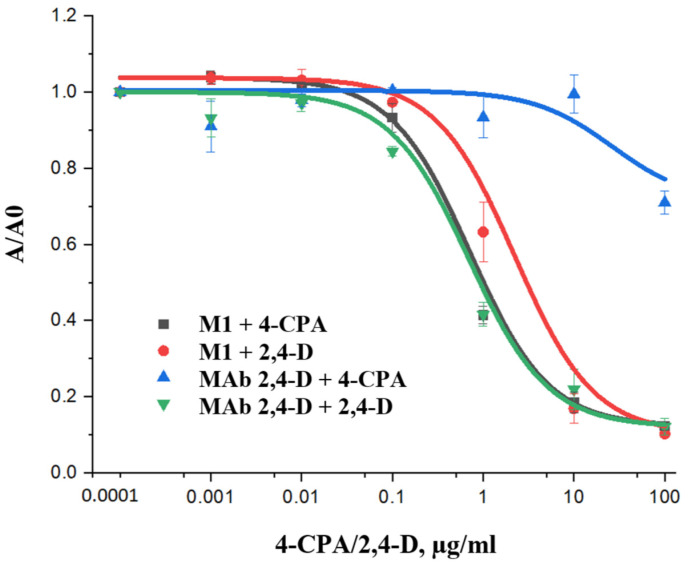
Graphs of the cross-reaction of M1 and MAb 2,4-D with 4-CPA and 2,4-D.

ELISA was performed according to the following protocol. Fifty µL of 4-CPA and 2,4-D were dispensed into 50 µL wells, and 4-CPA and 2,4-D were added at various concentrations and incubated for 1 h at 37 °C, with stirring (600 rpm), then washed 3 times with a PBS solution with 0.1% Tween; 100 μL of the working solution of the anti-species conjugate was added to the wells (sheep polyclonal antibodies to mouse IgG, labeled with peroxidase 1:20,000), incubated for 30 min at 37 °C, with stirring (600 rpm), then washed 5 times with a PBS solution with 0.1% Tween; 100 μL of TMB was added followed by incubation for 15 min in the dark at 37 degrees; the reaction was stopped with a solution of 5% sulfuric acid. The graphs are shown in Figure 1; the IC50 results are presented in Table 1.

A comparison of the experimental data (Table 1, Figure 1) revealed a striking difference in the specificity of the two antibody types. For clone M1 (anti-4-CPA), the half-maximal inhibitory concentrations (IC50) for 4-CPA and 2,4-D were comparable, indicating its inability to distinguish between these two structurally related compounds. Meanwhile, MAb2,4-D antibodies exhibit exceptional selectivity: their affinity for the target 2,4-D remains high, while cross-reactivity with 4-CPA is not detected within the detection limits of the assay.

### 2.4. Preparation and Purification of 4-CPA Tracers with Fluorescent Labels EDF (4-CPA-EDF) and 6-AHF (4-CPA-6-AHF)

The fluorescent label ethylenediaminefluoresceinisothiocyanate (EDF) was synthesized as described in a previous study [37]. First, 2.2 mg of 4-CPA (10 μmol) was dissolved in DMF, and 4.2 mg of DCC (20 μmol) and 2.3 mg of NHS (20 μmol) were added to the solution [36]. The reaction mixture was then incubated for 18 h in the dark with constant stirring. The mixture was then centrifuged for 5 min at 4000× *g* in an Eppendorf MiniSpin centrifuge (Eppendorf AG, Hamburg, Germany), and the supernatant was divided in half. Next, 1.8 mg (5 μmol) of EDF was added to one portion and 2.2 mg (5 μmol) of 6-AHF to the other and 20 µL triethylamine (TEA). 4-CPA-EDF and 4-CPA-6-AHF tracers were isolated by TLC in a CHCl_3_:CH_3_OH (4:1 by volume) system. The tracer bands were eluted from the chromatographic plate with methanol. Tracer concentrations were determined using the molar absorption coefficient of fluorescein, ε_491_ = 8.7 × 10^4^ M^−1^ cm^−1^ [31]. Typically, the yield during tracer synthesis is 30–40% of the starting material (fluorescein).

### 2.5. High-Performance Liquid Chromatography with High-Resolution Mass Spectrometry

The target compounds 4-CPA-EDF and 4-CPA-6-AHF, purified by TLC, were diluted 10-fold with methanol. The resulting samples were analyzed by HPLC-HRMS. The system included a Dionex Ultimate 3000 liquid chromatograph coupled with a Q Exactivehybrid high-resolution mass spectrometer (Thermo, Bremen, Germany). A HypersilGoldaQ (2.1 × 100 mm, 3 μm) analytical column (Thermo, Bremen, Germany) was used as the stationary phase. Mobile phase A consisted of 0.1% formic acid in 5% acetonitrile aqueous solution, and mobile phase B consisted of 0.1% formic acid in acetonitrile. Chromatographic separation was performed at a flow rate of 0.50 mL/min with the following gradient profile: 0–1.0 min 5% B, 1.0–15.0 min 5–95.0% B, 15.0–18.0 min 95% B, 18.0–18.1 min 95–5% B, and 18.1–20.0 min 5% B. The HPLC column thermostat temperature was set to 40 °C, and the sample injection volume was 1 µL. Mass spectra were recorded using Full MS in the 100–1000 *m*/*z* range, as well as All Ions Fragmentation (AIF) mode at 90–900 *m*/*z* in both positive and negative ionization modes [33].

The mass analyzer resolution was 35,000 (for 200 *m*/*z*). Sheath, aux, and sweep gas parameters were set to 40, 25, and 3 arbitrary units, respectively. Nebulizer voltage was +4.1 kV and −3.5 kV for positive and negative ionization modes, respectively, and the capillary temperature was 350 °C. The S-lens voltage was 50 arbitrary units, and the source temperature was 200 °C. Data processing was performed using Xcalibur software version 4.6 (Thermo, Bremen, Germany).

### 2.6. 4-CPA FPIA

Selection of working solution of tracers and monoclonal antibody concentration was conducted. A series of dilutions of 4-CPA-EDF and 4-CPA-6-AHF tracers (0.31–22.5 nM) were made in 50 mM borate buffer, pH 8.5 (BB), supplemented with NaN_3_ (0.01%). Fluorescence intensity and fluorescence polarization were measured in the prepared solutions. Fluorescence intensity and fluorescence polarization were measured in the prepared solutions in a tube made of borosilicate glass, size 10 × 75 mm. A Sentry 200 portable fluorimeter (light source LED, detector–photomultiplier tube, λex = 485 and λem = 535 nm, Ellie LLC, Germantown, WI, USA) was used for fluorescence polarization measurements. Dependences of changes in fluorescence intensity and polarization on tracer concentrations were plotted.

Tracer working solutions were prepared in BB with a concentration of 5 nM. Next, 0.5 mL of tracer solutions was added to 0.5 mL of M1 solutions in BB, in which the final concentration range was 0.2–63.5 nM. The change in fluorescence intensity and polarization for the prepared solutions was measured after 15 min of incubation at room temperature (25 °C). Based on the experimental data, the dependences of changes in fluorescence polarization on M1 concentrations were plotted, and the IC50 values were calculated.

The FPIA protocol for 4-CPA determinations: tracer solutions (4-CPA-EDF, 5 nM, 0.5 mL) were added to a series of 4-CPA solutions (0.001–100,000 ng/mL, 0.5 mL) in borate buffer (BB) and thoroughly mixed by vortex. A monoclonal antibody solution (M1 7 μg/mL (46.7 nM), 0.05 mL) was then added. After 15 min of incubation of the reaction mixtures, the FP signal was measured. All experiments were duplicated.

The dependences of the fluorescence polarization signal on the 4-CPA concentration were plotted and approximated by a four-parameter logistic function:

(1) $$Y=B+\frac{A-B}{\left(1+{\left(\frac{x}{C}\right)}^{D}\right)}$$

where *x* is the analyte concentration, *y* is the FP value, *A* is the asymptotic maximum of the FP value, *B* is the asymptotic minimum (background value) of the FP value, *C* is the inflection point of the curve in the semi-logarithmic coordinates (equal to 50% inhibition of the changes of FP signal), and *D* is the slope of the curve at the inflection point.

The limit of detection (LOD) was calculated as three sigma rules according to IUPAC recommendations [34]. The detection range of IC20–IC80, that is, the reduction of the FP signal by 20–80%, was estimated in accordance with [38,39]. When storing the reagents for 6 months, no significant changes were observed in the parameters of the analysis performed using them. Tracer stability in methanol solution ranges more than 6 months.

### 2.7. Evaluation of Assay Results and Specificity

The specificity of FPIA was studied under optimal assay conditions (Section 2.5). Competitive FPIA was performed using the following acids: 2,4-dichlorophenoxyacetic acid (2,4-D), 3,4-dichlorophenoxyacetic acid (3,4-D), 2-methyl-4-chlorophenoxyacetic acid (MCPA), 2,4,5-trichlorophenoxyacetic acid (2,4,5-T), glyphosate, atrazine, and chlorpyrifos in the concentration range of 0.001–100,000 ng/mL. Cross-reactivity (CR) was estimated using Equation (2):

(2) $$CR=\frac{{IC50}_{4\text{-}CPA}}{IC50_{cr}}\times 100\%$$

where IC50_cr_ and IC50_4-CPA_ are the concentrations of the cross-reagent and 4-CPA that cause 50% inhibition of monoclonal antibody binding to the tracer.

### 2.8. Competitive FPIA and Spiked Lake Water Sample Testing

Before FPIA, lake water samples were spiked with 4-CPA to final concentrations of 2.5, 5.0, 10.0, 50.0, 200.0, and 500 ng/mL, which were tested at the HPLC and shown to be free of chlorophenoxyacetic acids. Before analysis, the samples were filtered using 0.22-μm syringe filters and stored at 4 °C. Then, spiked samples were checked by FPIA protocol 4-CPA as described in Section 2.5. The FPIA was assessed by the recovery test, Equation (3):

(3) $$Recovery\%=\frac{C_{found}}{C_{added}}\times 100\%,$$

where C_found_ and C_added_ are the found and added concentrations of 4-CPA, respectively.

### 2.9. Conjugation of Antibodies Against 2,4-D and BrCN-Activated Sepharose 4B

The conjugation was carried out according to the manufacturer’s protocol [36]. First, 0.50 g of BrCN-activated Sepharose 4B was weighed. Additives were removed by washing with 100 mL of 0.001 mM HCl, resulting in a 1 mL suspension. Then, 0.2 mg of antibodies specific for 2,4-D in 0.1 M NaHCO_3_ containing 0.5 M NaCl (pH 8.5) were added to the BrCN-Sepharose and incubated with stirring at room temperature for 2 h. The sorbent was transferred to a column and washed with three column volumes of the sorption buffer (0.1 M NaHCO_3_ pH 8.3 containing 0.5 M NaCl) to remove unbound antibodies. Subsequently, the sorbent was washed with three volumes of 0.1 M Tris-HCl (pH 8.0) and left at room temperature for 2 h. After blocking of unreacted groups by 0.1 M Tris-HCl buffer, pH 8.0, during incubation for 2 h, the sorbent was washed with three cycles of alternating pH buffers: first with 5 mL of 0.1 M acetic acid containing 0.5 M NaCl (pH 4.0), then with 5 mL of 0.1 M Tris-HCl containing 0.5 M NaCl (pH 8.0).

## 3. Results and Discussion

During screening of hybridoma clones generated against 2,4-dichlorophenoxyacetic acid (2,4-D) [35], clone M1 was identified, demonstrating unexpected selectivity. In contrast to the expected specificity for 2,4-D, this clone exhibited higher affinity for 4-chlorophenoxyacetic acid (4-CPA). This suggests that the epitope recognized by the paratope of this antibody is more complementary to the monochloro-substituted derivative. Therefore, clone M1 can be used to detect 4-CPA.

### 3.1. Preparation and Characterization of Fluorescent Tracers

**Figure 2:**
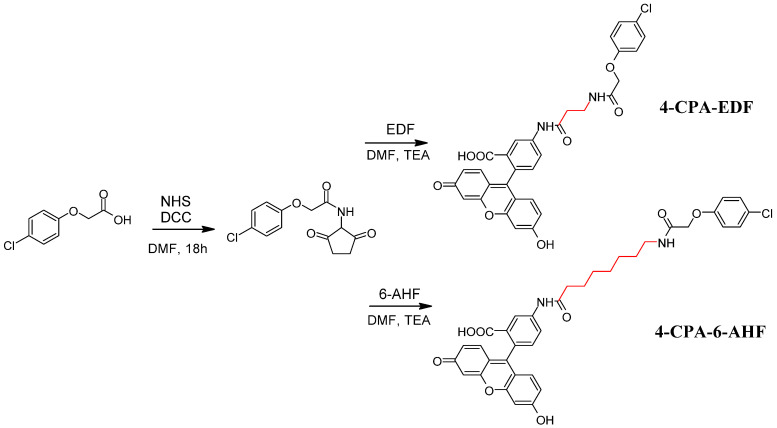
Synthesis of 4-CPA tracers with fluorescent labels EDF (4-CPA-EDF) and 6-AHF (4-CPA-6-AHF). Differences in the tracer structures (linker lengths) are highlighted in red.

To perform the FPIA determination of 4-CPA in samples, it was necessary to synthesize tracers with fluorescent labels. For this purpose, two fluorescein derivatives, EDF and 6-AHF, were used (Figure 1). The presence of functional amino groups in their structure allows for simple conjugation with 4-CPA, which contains a carboxyl group, to form an amide bond. For conjugation of 4-CPA with EDF and 6-AHF, activation with the carbodiimide method was used, which includes the step of interaction of DCC with the carboxyl group of 4-CPA to form a highly active intermediate O-acylsourea, which then reacts with the amino group of EDF or 6-AHF, forming an amide bond between 4-CPA and fluorescent compounds (Figure 2).

The reaction mixtures were then purified by TLC with a mobile phase of chloroform:methanol (4:1). This yielded a fraction with a retardation factor (Rf) of 0.9 for each tracer: 4-CPA-EDF and 4-CPA-6-AHF, respectively. The structures of the 4-CPA-EDF and 4-CPA-6-AHF tracers are shown in Figure 1. As can be seen from the figure, the fundamental difference between the tracers is the linkers of different lengths between fluorescein and 4-CPA: 4-CPA-EDF has a linker with two CH_2_ groups, while 4-CPA-6-AHF has six CH_2_ groups.

**Figure 3:**
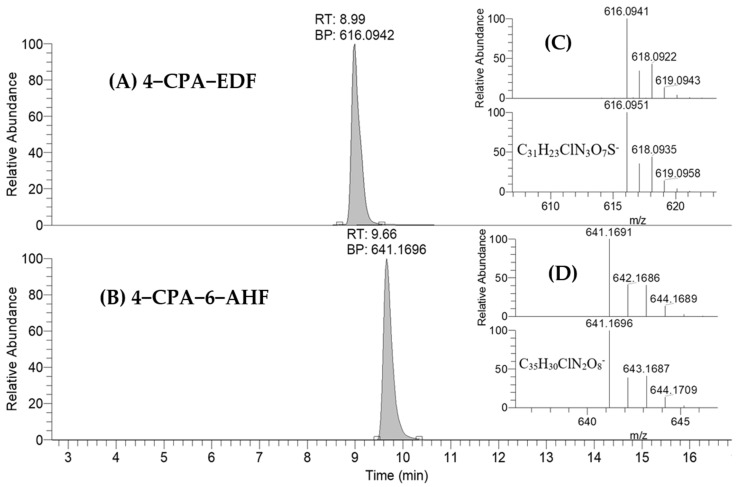
Mass chromatograms of the extracted ion currents with *m*/*z* 616.0951 (4-CPA-EDF (**A**)) and 641.1696 (4-CPA-6-AHF (**B**)), their isotope distributions (**C**) and (**D**), respectively, obtained in the total current recording mode of negatively charged ions, as well as the predicted isotope distributions for the corresponding gross formulas C_31_H_23_ClN_3_O_7_S^−^ and C_35_H_30_ClN_2_O_8_^−^.

**Figure 4:**
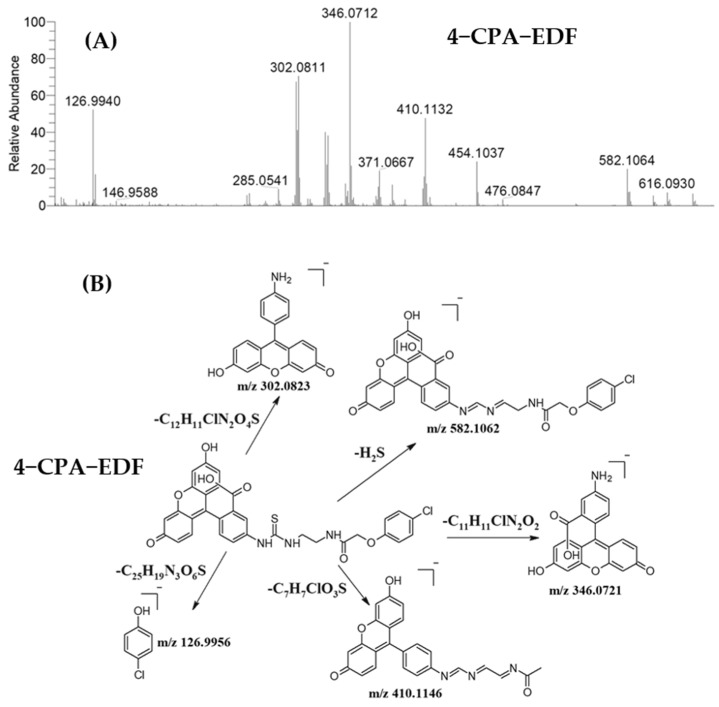
(**A**) MS/MS mass spectra of 4-CPA-EDF in negative ion mode. (**B**) Putative fragmentation pathways of the 4-CPA-EDF tracer.

**Figure 5:**
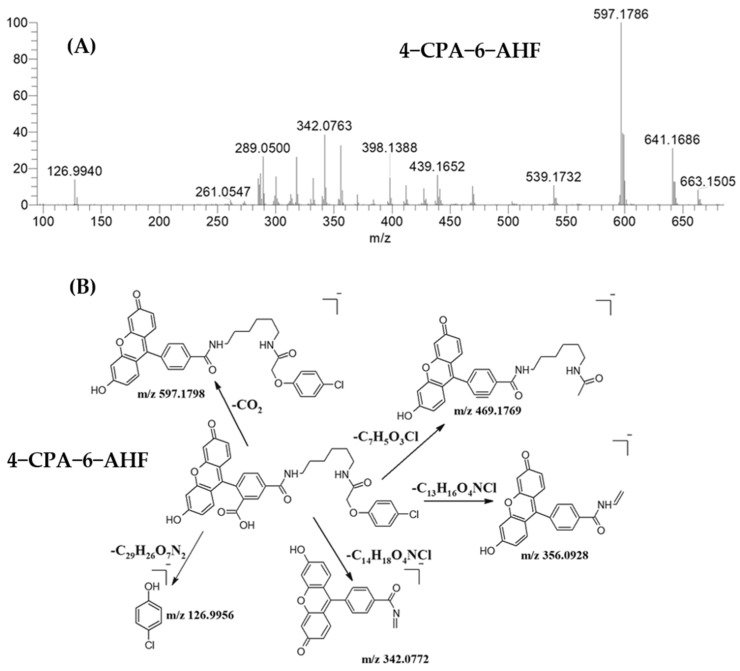
(**A**) MS/MS mass spectrum of 4-CPA-6-AHF in negative ion mode. (**B**) Putative fragmentation pathways of the 4-CPA-6-AHF tracer.

The structures of the target compounds, after TLC purification, were analyzed by high-performance liquid chromatography with high-resolution mass spectrometry (HPLC-HRMS). During the analysis, chromatograph mass spectrometric characteristics of the reagents were obtained in both positive and negative ionization modes, but the latter proved more informative. Therefore, this paper presents the following data obtained in negative ionization mode: experimental and predicted isotopic distributions in Figure 3; mass spectra obtained during ion fragmentation in Figure 4A and Figure 5A; and putative tracer fragmentation pathways in Figure 4B and Figure 5B.

As a result of HPLC-HRMS analysis of 4-CPA-EDF and 4-CPA-6-AHF compounds, the deprotonated molecular ions [M − H]^−^ were detected at *m*/*z* 616.0942 (C_31_H_23_ClN_3_O_7_S, −1.6 ppm) and 641.1696 Da (C_35_H_30_ClN_2_O_8_, 0.01 ppm) (Figure 4A,B). The obtained isotope distributions were consistent with the isotope distributions predicted for the indicated molecular formulas (Figure 3C,D), confirming the elemental compositions.

The MS/MS spectrum of 4-CPA-EDF (Figure 4A) reveals diagnostic fragment ions spanning the entire molecule. Ions at *m*/*z* 302.0811 (C_19_H_12_NO_3_, −0.23 ppm) and 346.0712 Da (C_20_H_12_NO_5_, 0.58 ppm) originate from the fluorescein core. The ion at *m*/*z* 410.1132 (C_24_H_16_N_3_O_4_, −0.81 ppm) corresponds to the fluorescein with the linker fragment, while *m*/*z* 582.1064 (C_31_H_21_ClN_3_O_7_, −0.09 ppm) represents the fluorescein-linker-4-CPA assembly. Critically, the presence of the 4-CPA residue is independently confirmed by the ion at *m*/*z* 126.9940 Da (C_6_H_4_ClO, −4.1 ppm). The main fragmentation pathways are summarized in Figure 4B.

The MS/MS spectrum of 4-CPA-6-AHF (Figure 5A) exhibits analogous fragmentation behavior (Figure 5B). Ions at *m*/*z* 342.0763 (C_21_H_12_NO_4_, 0.63 ppm) and 356.0920 Da (C_22_H_14_NO_4_, 0.75 ppm) correspond to fluorescein-containing fragments. The ion at *m*/*z* 469.1760 Da (C_28_H_25_N_2_O_5_, 0.43 ppm) arises from the fluorescein-linker-core with the carbonyl group derived from the 4-CPA fragment. The ion at *m*/*z* 597.1786 Da (C_34_H_30_N_2_O_6_Cl, −0.15 ppm) represents the fluorescein-linker-4-CPA assembly after neutral loss of CO_2_. As with 4-CPA-EDF, the characteristic 4-CPA fragment is observed at *m*/*z* 126.9940 Da (C_6_H_4_ClO, −4.1 ppm).

The presence of the 4-CPA signature fragment (*m*/*z* 126.9956) in both compounds, together with the complementary ions covering each structural motif, provides unequivocal evidence that the 4-CPA residue remains intact and covalently attached to the linker-fluorescein scaffold. The absence of unexpected fragment ions and the excellent match between observed and calculated *m*/*z* values (<5 ppm) rule out structural isomers and byproducts. Collectively, the MS, isotope distribution, and MS/MS data confirm the successful synthesis of both tracers.

**Figure 6:**
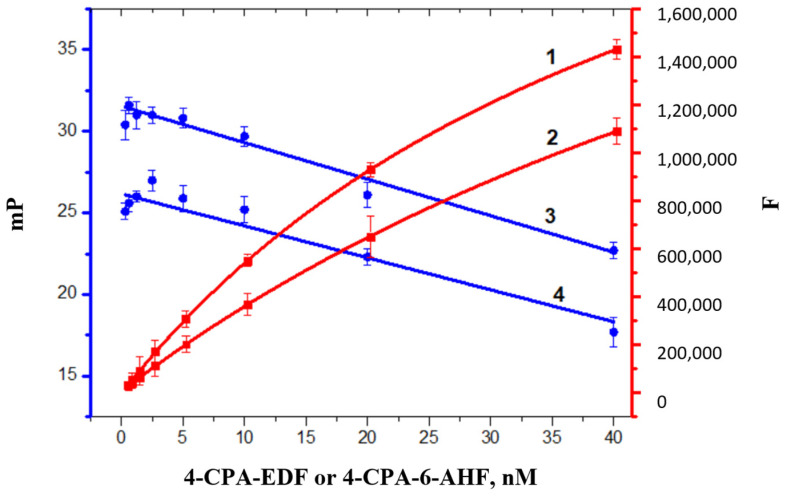
Changes in the fluorescence intensity signal of 4-CPA-6-AHF (1) and 4-CPA-EDF (2), fluorescence polarization of 4-CPA-EDF (3) and 4-CPA-6-AHF (4), at different concentrations (25 °C; BB; pH 8.5) (n = 3).

After synthesizing the compounds (4-CPA-EDF and 4-CPA-6-AHF), their fluorescence properties were studied. For this purpose, a dilution series of each tracer was prepared over a concentration range spanning several orders of magnitude (from 0.1 to 50 nM). For each sample, two parameters were simultaneously measured on a Sentry200 instrument: fluorescence intensity (F, in relative units) and fluorescence polarization (mP). The obtained data are presented in a graph (Figure 6), where the X-axis represents the tracer concentration, and the Y-axis represents the F (right) and mP (left) values. At low tracer concentrations (approximately 0.1 nM), the fluorescence intensity is close to the instrument noise level and increases slowly. As the concentration increases to 1–10 nM, a nearly linear increase in F is observed, indicating the absence of noticeable concentration quenching. At concentrations above 10 nM, linearity is disrupted, and the increase in intensity decreases due to secondary effects (e.g., the internal filter effect or aggregation of tracer molecules). Similarly, at low tracer concentrations, the polarization signal is unstable and fluctuates significantly due to the low signal-to-noise ratio. In the concentration range from 1 to 10 nM, the mP value remains virtually constant, i.e., independent of the tracer concentration. This means that tracer molecules in this range behave as individual, unassociated particles with the same rotational correlation time. Above 10 nM, polarization decreases due to intermolecular interactions or aggregation. For correct performance of the fluorescence polarization immunoassay (FPIA), the polarization should stay stable and not depend on uncontrolled tracer concentration fluctuations. Therefore, the optimal concentration range is one where mP is constant (unchanging within the measurement error) and fluorescence intensity increases linearly with concentration. This range corresponds to 1–10 nM for both synthesized tracers. For further work (establishing FPIA and calibration curves), a concentration of 5 nM—the center point of this plateau—was chosen. This concentration ensures sufficient fluorescence intensity (high signal-to-noise ratio), polarization signal stability, and minimal sensitivity to random errors during dilution of working solutions.

### 3.2. Antibody Testing

To select the working concentration of specific monoclonal antibody M1, we studied its binding to 4-CPA-EDF and 4-CPA-6-AHF tracers and compared its affinity with the nonspecific monoclonal antibody M2. To 1 mL of 4-CPA-EDF or 4-CPA-6-AHF tracer with a concentration of 2.5 nM, 0.05 mL of M1 and different concentrations of M2 were added so that the final concentrations of antibodies in the solution were 0.2–63.5 nM, and the changes in mP values were measured after 15 min. Then, the proportion of the bound fraction (Fb), which is the ratio of the bound tracer to the total concentration of the tracer, was calculated (Equation (4)):

(4) $$Fb=\frac{mP-{mP}_{free}}{{mP}_{max}-{mP}_{free}}$$

where mP is the measured FP signal, ${mP}_{free}$ is the FP of free tracer, and ${mP}_{max}$ is the FP at maximum binding of antibody and tracer.

**Figure 7:**
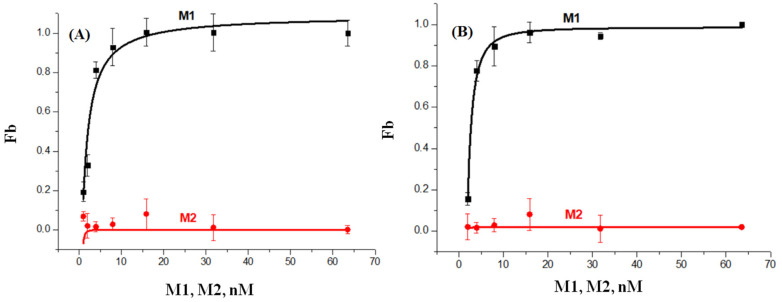
Changes in the proportion of the bound fraction (Fb) for tracers (**A**) 4-CPA-EDF (2.5 nM) and (**B**) 4-CPA-6-AHF upon interaction with antibodies M1 and M2 at 25 °C, pH 8.5 (n = 3).

The results of the dependence of Fb on the concentration of antibodies in the solution are shown in Figure 7.

These results demonstrate that the interaction of antibody M1 with both tracers results in a sharp increase in fluorescence polarization (mP), even at low antibody concentrations. The FP signal plateaus at an M1 antibody concentration of 15.8 nM for both tracers. Moreover, the addition of nonspecific antibody M2 to the 4-CPA-EDF and 4-CPA-6-AHF tracers did not cause a change in the fluorescence polarization signal, indicating the specificity of binding of the tracers to M1 and M2. For further study, concentrations of antibody M1 were selected that yielded a bound fraction (Fb) of 0.5 [36]. The final concentrations of antibody in solution for tracers 4-CPA-EDF and 4-CPA-6-AHF consist of 2.2 ± 0.1 and 3.2 ± 0.3 nM, respectively.

### 3.3. Development of 4-CPA FPIA

**Figure 8:**
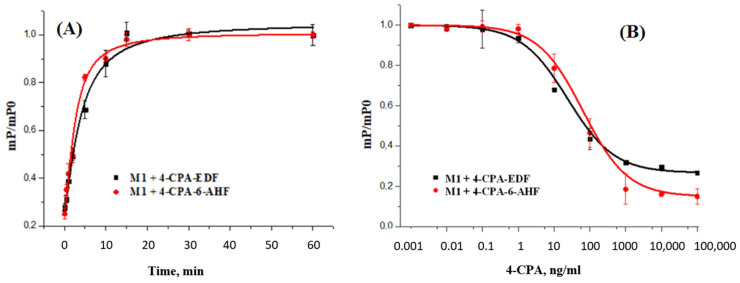
(**A**) Kinetics of the fluorescence polarization change at interaction 4-CPA-EDF and 4-CPA-6-AHF with specific MAb (2.2 nM and 3.2 nM, respectively, n = 3) (**B**) Normalized dependences of the change in fluorescence polarization signals (mP/mP0) on the concentration of 4-CPA for 4-CPA-EDF and 4-CPA-6-AHF tracers with antibody M1. The final concentration of the tracers was 2.5 nM, and the M1 concentration for tracers 4-CPA-EDF and 4-CPA-6-AHF was 2.2 and 3.2 nM, respectively (n = 3).

Initially, we studied the kinetics of binding of M1 antibodies to the tracers 4-CPA-EDF and 4-CPA-6-AHF and showed that equilibrium in the system is established after 15 min (the results are presented in Figure 8A), and the fluorescence polarization signal is maintained for 60 min. Therefore, 15 min was chosen for the analysis time. To select a tracer for developing a sensitive competitive FPIA and compare it with the normalized mP/mP0 dependences for 4-CPA-EDF and 4-CPA-6-AHF (Figure 8), we obtained dependences of the FP signal change on the 4-CPA concentration.

The analytical characteristics of the dependences of the normalized FP signal change for the determination of 4-CPA for the tracers 4-CPA-EDF and 4-CPA-6-AHF are presented in Table 1. Thus, Table 2 demonstrates that a more sensitive FPIA can be obtained with the 4-CPA-EDF tracer, which has a shorter spacer.

**Figure 9:**
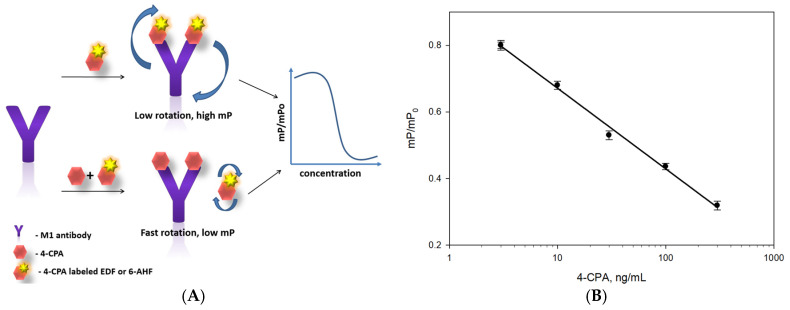
(**A**) Competitive format FPIA for 4-CPA. (**B**) Detection range for 4-CPA determination by FPIA. The final reagent concentrations are [4-CPA-EDF] = 2.5 nM, [M1] = 2.2 nM (BB; pH = 8.5). Y = (0.91 ± 0.02) − (0.24 ± 0.01) × X (R2 = 0.98; n = 3).

The resulting calibration curve for the 4-CPA assay and its linear approximation (in semi-logarithmic axes) are shown in Figure 9. The developed FPIA method enables the determination of 4-CPA with a detection limit of 1.2 ng/mL (the detection limit was calculated as three sigma rules [34]). The range of detectable concentrations is quite wide: 2.3–300 ng/mL. This manuscript presents a series of implemented experiments based on such a design. It should be noted that repeatedly obtaining the calibration dependences did not cause reliable changes in fitting functions and the calculated analytical parameters of the testing.

### 3.4. FPIA Specificity Study

To determine the specificity of the developed FPIA, cross-reactivity (CR) testing was conducted with compounds structurally similar to 4-CPA and non-related pesticides. CR (%) was calculated using Equation (3) and shown in Table 3. These results demonstrate that this test system can detect 2,4-D in addition to 4-CPA. Based on this cross-reactivity, a dual determination of 4-CPA and 2,4-D is possible.

### 3.5. Determination of 4-CPA by FPIA in Lake Water by the Recovery Test

Validation of the developed test system was performed using the add-and-find method on a real water sample from an open reservoir. For this purpose, seven lake water samples, previously tested for the 4-CPA herbicide by HPLC, were prepared and analyzed at concentrations of 5, 10, 50, and 200 ng/mL using the method that corresponds to Section 2.5. The 4-CPA recovery percentage was determined using the calibration curves obtained above (Figure 8). The accuracy of the method is assessed by the recovery percentage. The test results are presented in Table 4. The 4-CPA recovery percentage in lake water ranged from 98% to 110%.

Thus, it was demonstrated that the FPIA method can be used to detect 4-CPA in water in a wide range of concentrations.

### 3.6. Dual Assay for 4-CPA and 2,4-D

2,4-D is a fairly widely used pesticide, and the developed FPIA method for 4-CPA has a significant overlap with 2,4-D (Table 2). Therefore, a dual assay was developed to quantify 4-CPA using the already established FPIA method for 2,4-D [36]. Namely, it is possible to determine the concentration of 4-CPA in water using a dual test. To test this hypothesis, samples were prepared and spiked with different amounts of 2,4-D and 4-CPA (Table 5) and tested using a dual assay: FPIA for 2,4-D as described in [36] and for 4-CPA, as described in this study.

The following procedure of data processing was applied for this dual testing [40,41].

The 4-CPA test system detects 4-CPA with a response of A to its single concentration, but when 2,4-D is present in the sample, it also produces a response of 0.48 (Table 4) of the specific response to 4-CPA, i.e., 0.48 × A to the single concentration of 2,4-D. The 2,4-D test system detects 2,4-D with a response of B to its single concentration, but if the sample contains 4-CPA, it also produces a response of 0 (zero) to the specific response to 2,4-D, i.e., 0 × B to the single concentration of 4-CPA. A sample containing 4-CPA at a concentration of X and 2,4-D at a concentration of Y was taken. When the sample was tested, the 4-CPA test system produced a response of C1, and the 2,4-D test system produced a response of C2.

C1 = X × A + Y × 0.48 × A(5)

C2 = X × 0 × B + Y × B(6)

To simplify the calculations, we divide Equation (4) by the coefficient A and Equation (5) by the coefficient B and introduce the term apparent concentration D1 for substance 4-CPA and apparent concentration D2 for 2,4-D.

Then the system of equations is simplified to the following:

D1 = X + Y × 0.48(7)

D2 ≈ Y(8)

As shown in Table 5, the recovery ranged from 99 to 151%, indicating the ability to accurately determine the concentration of 4-CPA in water samples.

### 3.7. Depletion of Water Samples Containing 4-CPA and 2,4-D

To address cross-reactivity with the common herbicide 2,4-dichlorophenoxyacetic acid (2,4-D), a previously described approach was developed and successfully implemented. This approach involves simultaneously conducting two separate FPIAs—one for 2,4-D and one for 4-CPA—followed by mathematical decomposition of the two analyte concentrations based on the measured binding values. While this method is functional, it inevitably requires two measurements and subsequent calculations. To avoid the need for parallel testing and mathematical decomposition, a second, simpler strategy was employed: selective removal of 2,4-D from sample matrices using affinity chromatography columns with immobilized anti-2,4-D antibodies prior to FPIA for 4-CPA. This alternative offers a cleaner, one-step solution to the cross-reactivity problem, eliminating the complexity of dual analysis and algebraic deconvolution.

In our study, we attempted to separate 2,4-D from the test samples using affinity chromatography and subsequent 4-CPA determination by FPIA. Using a standard method, antibodies specific for 2,4-D were conjugated to BrCN-activated Sepharose 4B (MAb2,4-D-Seph), and this affinity carrier was used to separate 2,4-D from spiked water samples.

#### 3.7.1. Testing Samples Containing Only 2,4-D

First, we tested whether the sorbent would bind 2,4-D at different concentrations in water samples. For this purpose, we prepared water samples containing 2,4-D at concentrations of 30, 60, and 120 ng/mL. The pH of the samples was adjusted to 9.0. Then, 0.5 mL of each sample was passed through the column, and 1 mL was collected. Taking dilution into account, this resulted in samples with 2,4-D concentrations of 15, 30, and 60 ng/mL. These concentrations were chosen considering that the detection limit for 2,4-D in water is 30 ng/mL; accordingly, concentrations twice as high and lower were additionally tested.

**Figure 10:**
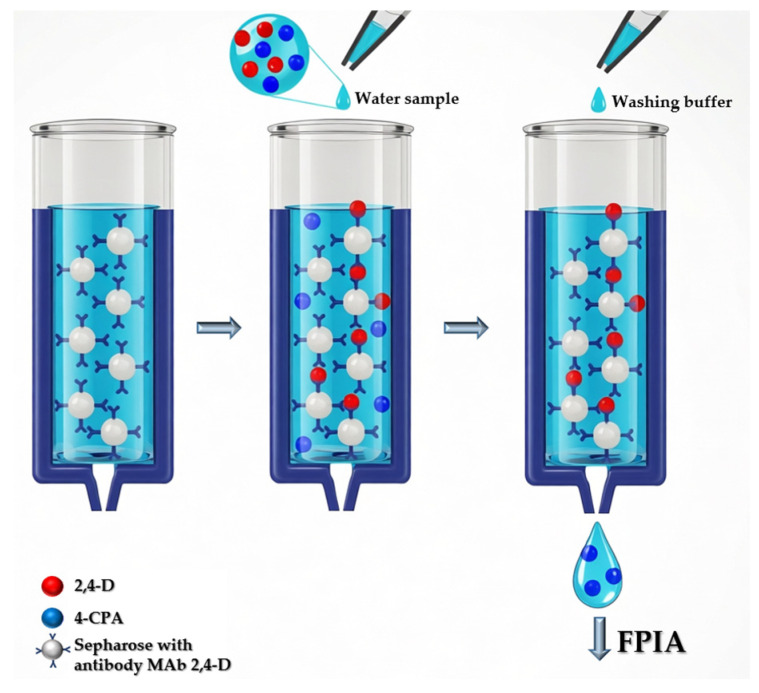
Preparation of water samples using affinity column MAb 2,4-D-Seph.

The influence of residual amounts of 2,4-D analyzed as follows. To 500 µL of borate buffer or to the 2,4-D samples of different concentrations (15, 30, and 60 ng/mL), 500 µL of a ready-made 5 nM solution of the tracer 4-CPA-6-AHF was added, followed by the working solution of M1 antibodies specific to 4-CPA. Fluorescence polarization of the zero sample (containing no 2,4-D) and fluorescence polarization of samples with different concentrations of 2,4-D after column purification showed almost no difference, indicating that all 2,4-D in the samples was adsorbed on the column (Figure 10).

#### 3.7.2. Testing Samples Containing 2,4-D and 4-CPA at Different Concentrations

Four samples were prepared, each containing 2,4-D at a concentration of 60 ng/mL and 4-CPA at concentrations of 10, 100, and 1000 ng/mL. Water samples pre-spiked with the herbicides 4-CPA and 2,4-D were adjusted to pH 9.0 and passed through a column packed with MAb 2,4-D-Seph, as described in experiment A. Fluorescence polarization of the resulting samples was measured in the same manner as in experiment A. The results are presented in Table 6.

As shown by the data in Table 6, 2,4-D was completely adsorbed on the column and did not interfere with the determination of 4-CPA. As the concentration of 4-CPA increases, the fluorescence polarization decreases, indicating the possibility of accurate determination of 4-CPA in water samples.

## 4. Conclusions

Highly specialized immunoreagents have been successfully developed, including monoclonal antibodies targeting 4-chlorophenoxyacetic acid (4-CPA). Additionally, two 4-CPA tracers were synthesized and purified, featuring fluorescent labels, EDF and 6-AHF. The structural integrity of these compounds was confirmed through advanced techniques such as high-performance liquid chromatography coupled with high-resolution mass spectrometry (HPLC-HRMS), ensuring their suitability for further applications. The length of the linker between the fluorophore and the hapten is thought to influence FPIA sensitivity through three main mechanisms: 1. Antibody accessibility. A short linker (as in EDF) keeps the fluorophore close to the hapten epitope. Upon binding to the antibody, the fluorophore may be partially shielded by amino acid residues in the binding pocket, reducing the efficiency of quenching or polarization change. A long linker (6-AHF) places the fluorophore further from the antibody surface, reducing steric hindrance and allowing freer rotation of the unbound tracer. Upon binding, however, rotation is more constrained due to the remote “anchor.” However, an excessively long linker can increase flexibility and partial internal rotation, which reduces polarization gain. 2. Rotational diffusion and correlation time. According to the Perrin equation, polarization (mP) depends on the ratio of the fluorophore lifetime (τ) to the rotational correlation time (θ). For a short linker, the fluorophore is rigidly bound to the hapten, and upon binding to the antibody, tracer rotation is completely determined by the large complex (antibody-hapten-fluorophore), resulting in high polarization. For a long linker, the fluorophore may retain some local mobility even in the bound state (segmental mobility), which reduces the effective rotational correlation time and, consequently, lowers the maximum polarization. This may explain differences in the dynamic range of the signal. 3. Fluorescence lifetime and nonradiative processes. Linker length can influence the fluorophore microenvironment (polarity, viscosity, proximity to antibody quenching). In our experiments, 6-AHF (a long linker) showed a higher increase in polarization upon binding, indicating less internal rotation and/or greater efficiency in transferring rotational constraints. It is possible that the 6-AHF linker has optimal rigidity, while EDF (a short linker) prevents the fluorophore from escaping steric clashes with the antibody.

Although a direct correlation between “the longer the linker, the higher the sensitivity” is not observed (sensitivity is determined by a balance between flexibility, accessibility, and lifetime preservation), the difference between EDF and 6-AHF is explained by a combination of these factors [39].

In the pursuit of creating an effective and optimized fluorescence polarization immunoassay (FPIA) for quantifying 4-CPA in aquatic environments, optimal concentrations of the immunoreagents were meticulously selected. The assay demonstrated a remarkable detection limit of 1.2 ng/mL, with a linear range spanning from 2.3 to 300 ng/mL, making it a highly sensitive tool for monitoring this herbicide.

FPIA significantly outperforms instrumental methods such as GLC (LOD 50 ng/mL) and SPE-GC-MS in its upper range and is on par with QuEChERS-LC-MS/MS (LOD < 10 ng/mL). Among immunochemical methods, FPIA is more sensitive than conventional ICA (LOD 50 ng/mL) and approaches the sensitivity of ELISA, though a more sensitive ICA format has been reported (LOD 0.2 ng/mL). However, FPIA’s key advantages are its rapidity (15 min) the absence of sample preparation and time-consuming manipulations, making it suitable for point-of-sample screening.

The developed FPIA method is more than just another immunoassay. To our knowledge, this is the first quantitative FPIA for 4-CPA in water that delivers results within 15 min with a detection limit of 1.2 ng/mL. Its operational characteristics surpass all instrumental methods listed in Table 1, except for the most complex and expensive LC-MS approaches. In terms of the balance between sensitivity, time, cost, and simplicity, FPIA has no direct competitors. It combines the analytical reliability of ELISA, the speed of ICA, and the quantitative performance of chromatography. This method can be recommended for implementation in monitoring services. Table 7 summarizes the comparative characteristics of the methods for determining 4-CPA.

The fluorescence polarization immunoassay (FPIA) system utilizing the 4-CPA-EDF tracer in conjunction with the M1 antibody displayed notable cross-reactivity with other herbicides, specifically 2,4-D, while showing minimal interaction with 3,4-D and MCPA. This characteristic is crucial for ensuring specificity in environmental assessments. In addition, the recovery test showed that the recovery percentage varied from 98% to 112%. Such high recovery rates indicate the reliability and accuracy of the assay, positioning it as a recommended method for the rapid detection of 4-CPA in various water bodies.

Because of the high cross-reactivity of FPIA for 4-CPA with the prevalent herbicide 2,4-D, two novel strategies were devised and successfully implemented. The first approach relies on the concurrent execution of two separate FPIAs—for 2,4-D and 4-CPA—followed by mathematical resolution of the two analyte concentrations from the measured binding values. While functional, this method inevitably requires dual measurements and post hoc calculation. Motivated by the desire to circumvent the need for parallel testing and mathematical processing, a second, more straightforward strategy was pursued: the selective removal of 2,4-D from sample matrices using affinity chromatography columns with immobilized anti-2,4-D antibodies prior to FPIA for 4-CPA. This alternative offers a cleaner, single-step resolution of the cross-reactivity issue, eliminating the complexity of dual analyses and algebraic deconvolution. Both proposed methodologies appear highly promising for overcoming the inherent limitations of traditional immunoassays when faced with significant cross-reactivity among structurally analogous compounds.

## Figures and Tables

**Table 1:** Values of IC50 for M1 and MAb 2,4-D with 4-CPA and 2,4-D.

µg/mL	M1 + 4-CPA	M1 + 2,4-D	MAb 2,4-D + 2,4-D
IC50	0.71	2.22	0.70

**Table 2:** FPIA analytical characteristics for the determination of 4-CPA in solution (n = 3).

Tracer/MAb	LOD, ng/mL	IC20, ng/mL	IC50, ng/mL	IC80, ng/mL
4-CPA-6-AHF/M1	6.3 ± 0.6	10.5 ± 1.8	50 ± 2.2	344.2 ± 5.0
4-CPA-EDF/M1	1.2 ± 1.2	2.3 ± 0.4	15.2 ± 1.4	301.6 ± 3.1

**Table 3:** Cross-reactivity of FPIA for 4-CPA (n = 3).

Substances	Structure	CR, %
4-Chlorophenoxyacetic acid (4-CPA)	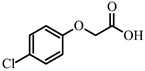	100
2,4-Dichlorophenoxyacetic acid (2,4-D)	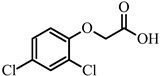	48
3,4-Dichlorophenoxyacetic acid (3,4-D)	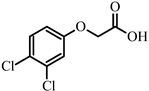	0.2
2-Methyl-4-chlorophenoxyacetic acid (MCPA)	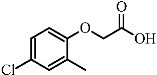	6
2,4,5-Trichlorophenoxyacetic acid (2,4,5-T)	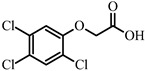	25
Glyphosate	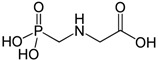	<0.1
Atrazine	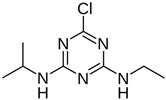	<0.1
Chlorpyrifos	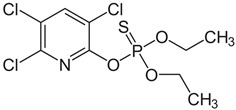	<0.1

**Table 4:** Results of the recovery test for 4-CPA in water by FPIA (n = 3).

№	Added, ng/mL	Found, ng/mL	Recovery %
1	5	5.1 ± 0.23	102 ± 3.1
2	10	9.8 ± 0.52	98 ± 6.6
3	50	55.2 ± 4.99	110 ± 9.5
4	200	205.2 ± 7.6	103 ± 4.4

**Table 5:** Results of the recovery test for 4-CPA by FPIA in the presence 2,4-D (n = 3).

№	Added, ng/mL		Found by FPIA, ng/mL		Calculated4-CPA, ng/mL (X)	Recovery 4-CPA, %
	4-CPA	2,4-D	4-CPA * (C1)	2,4-D ** (C2 = y)		
1	2.5	5	5.4 ± 0.6	5.2 ± 0.3	2.9 ± 0.3	116 ± 2.5
2	5	10	12.4 ± 1.2	11.3 ± 0.2	6.9 ± 1.1	138 ± 4.3
3	10	20	25.1 ± 2.7	19.5 ± 0.3	15.7 ± 1.5	151 ± 6.5

* detected pesticide concentration by the 4-CPA-FPIA system ** detected pesticide concentration by the 2,4-D-FPIA system.

**Table 6:** Recovery test to determine 4-CPA in water samples containing 30 ng/mL of 2,4-D and different concentrations of 4-CPA using FPIA after depletion of 2,4-D on an affinity column (MAb 2,4-D-Seph).

Sample	Added		mP/mP_0_	Found 4-CPA, ng/mL	Recovery %
	2,4-D, ng/mL	4-CPA, ng/mL			
1	0	0	1	0	ND *
2	30	0	0.98 ± 0.01	0	ND
3		5	0.83 ± 0.03	5.5 ± 0.1	110.0 ± 2.0
4		50	0.61 ± 0.02	52.0 ± 2.0	104.4 ± 1.5
5		500	0.45 ± 0.05	518.0 ± 5.0	103.6 ± 3.0

* ND—not determined.

**Table 7:** Comparison of methods for determining 4-CPA.

Assay Format	Analyte-Binding Reactant	LOD, ng/mL	Assay Time	Sample Tested	Reference
FPIA	MAb	1.2	15 min	Water	[This work]
ELISA	MAb	0.84	2–3 h	Bean sprout	[19]
ICA	MAb	50	15 min	Bean sprout	[19]
QuEChERS-LC-MS/MS		<10	40 min	Peanut products; popcorn	[42]
SPE-GC-MS		From 5 to 340	40–60 min	Bean sprout	[12]
GLC		50	-	Water	[26]

## Data Availability

Data are available upon request from authors.

## Abbreviations

The following abbreviations are used in this manuscript:

2,4-D	2,4-chlorophenoxyacetic acid
2,4,5-T	2,4,5-trichloroacetic acid
3,4-D	3,4-dichlorophenoxyacetic acid
4-CPA	4-chlorophenoxyacetic acid
6-AHF	aminohexylaminocarbonylfluorescein
DCC	N,N’-dicyclohexylcarbodiimide
EDF	ethylenediamine fluorescein thiocarbamate
FPIA	fluorescence polarization immunoassay
MCPA	2-methyl-4-chlorophenoxyacetic acid
NHS	N-hydroxysuccinimide
